# The impact of a meal, snack, or not eating during the night shift on simulated driving performance post-shift

**DOI:** 10.5271/sjweh.3934

**Published:** 2020-12-16

**Authors:** Charlotte C Gupta, Stephanie Centofanti, Jillian Dorrian, Alison M Coates, Jacqueline M Stepien, David Kennaway, Gary Wittert, Leonie Heilbronn, Peter Catcheside, Georgia A Tuckwell, Daniel Coro, Dilushi Chandrakumar, Siobhan Banks

**Affiliations:** 1 Appleton Institute, School of Health, Medical and Applied Sciences, Central Queensland University, Adelaide, South Australia, Australia; 2 University of South Australia Online, Adelaide, South Australia, Australia; 3 Behaviour-Brain-Body Research Centre, UniSA Justice & Society, University of South Australia, Adelaide, South Australia, Australia; 4 Alliance for Research in Exercise, Nutrition and Activity Research Concentration, UniSA Allied Health & Human Performance, University of South Australia, Adelaide, South Australia, Australia; 5 Robinson Research Institute and Adelaide School of Medicine, University of Adelaide, Adelaide, South Australia, Australia; 6 Discipline of Medicine, Adelaide Medical School, University of Adelaide, Adelaide, South Australia, Australia; 7 Adelaide Institute for Sleep Health, College of Medicine and Public Health, Flinders University, Adelaide, South Australia, Australia

**Keywords:** cognition, meal pattern, meal timing, nocturnal eating, shift work

## Abstract

**Objective:**

The commute home following a night shift is associated with an increased risk for accidents. This study investigated the relationship between food intake during the night shift and simulated driving performance post-shift.

**Methods:**

Healthy non-shift working males (N=23) and females (N=16), aged 18–39 years (mean 24.5, standard deviation 5.0, years) participated in a seven-day laboratory study and underwent four simulated night shifts Participants were randomly allocated to one of three conditions: meal at night (N=12; 7 males), snack at night (N=13; 7 males) or no eating at night (N=14; 9 males). During the night shift at 00:30 hours, participants either ate a large meal (meal at night condition), a snack (snack at night condition), or did not eat during the night shift (no eating at night condition). During the second simulated night shift, participants performed a 40-minute York driving simulation at 20:00, 22:30, 01:30, 04:00, and 07:30 hours (similar time to a commute from work).

**Results:**

The effects of eating condition, drive time, and time-on-task, on driving performance were examined using mixed model analyses. Significant condition×time interactions were found, where at 07:30 hours, those in the meal at night condition displayed significant increases in time spent outside of the safe zone (percentage of time spent outside 10 km/hour of the speed limit and 0.8 meters of the lane center; P<0.05), and greater lane and speed variability (both P<0.01) compared to the snack and no eating conditions. There were no differences between the snack and no eating conditions.

**Conclusion:**

Driver safety during the simulated commute home is greater following the night shift if a snack, rather than a meal, is consumed during the shift.

The commute home following a night shift is a time of great risk for driver safety (1, 2), with shift workers frequently reporting falling asleep at the wheel and experiencing near-misses (3, 4). For shift workers, this may be due to the increase in homeostatic sleep pressure after being awake for a long period of time overnight (5), which may be exacerbated by the length of the commute (6) and an increase in morning road users at the time of the post-shift commute (7).

Several strategies are commonly utilized to improve alertness for the commute home, including caffeine, playing loud music, napping, bright light, and stopping for breaks (7–11). While these strategies may subjectively improve alertness, empirical evidence does not support the use of all of these countermeasures for improving driving performance (9, 12). As the need for shiftwork increases, so too does the need to identify new countermeasures and strategies to reduce the impacts of fatigue on the commute home (8, 13). One way of identifying new countermeasures is to consult the strategies and behaviors that shift workers already report on-shift. Altered eating behaviors during the night compared to day shift is one such behavior that that is common amongst shift workers (14) and could have implications for performance on-shift.

Our research has previously shown that restricting the amount of food consumed during a simulated night shift improved driving performance during the night and reduced subjective sleepiness (15). It is unknown if this impairment would extend to 07:30 hours when workers are typically driving home and whether reducing or limiting food during the night could improve driving performance. This study investigated the impact of eating a meal, snack, or not eating during the night shift on driving performance during a simulated post-night shift commute home.

## Methods

### Study design

The study was a seven-day experimental, three-condition, between-group design conducted in the windowless, sound attenuated, and time-isolated Sleep and Chronobiology Laboratory at the University of South Australia (UniSA). All participants gave written informed consent and the UniSA Research Ethics Committee approved the study (#0000033621), which was registered with the Australian New Zealand Clinical Trials Registry (ANZCTR12615001107516). This manuscript reports secondary outcome data from a larger study and additional measures have been published elsewhere (15, 16).

### Participants

Healthy, non-shift working participants were recruited from the general population via flyers and website postings. All participants were 18–45 years, within a body mass index (BMI) range of 18.5–27kg/m^2^, non-smokers, had no chronic medical illnesses, were not shift workers, and had habitual sleep patterns that included going to bed between 21:00–00:00 hours and waking between 06:00–09:00 hours [for specific exclusion criteria, please see (15, 16)].

### Protocol design

**Figure 1:**
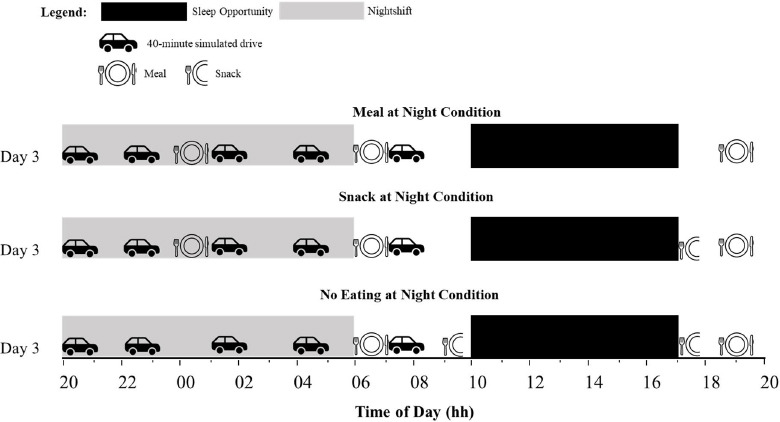
Protocol diagram for day 3 of the 7-day in-laboratory simulated nightshift protocol. Upper panel shows the Meal at Night condition, the middle panel shows the Snack at Night condition, and the lower panel shows the No Eating at Night condition. Black bars indicate the sleep period, and grey bars indicate the nightshift. Car icons indicate the performance times (20:00, 22:30, 01:30, 04:00, and 07:30 hours).

Ambient temperature was controlled [mean 22, standard deviation (SD) 1 °C] and light intensity was <100 lux at eye-level during wake and <0.03 lux during sleep. The protocol of the seven-day simulated shift work study has been described in detail elsewhere (16), and this study describes results from night shift 2 of the larger study. Figure 1 shows the protocol for night shift 2 (day 3).This was the only morning that a 07:30 hour simulated drive was included as an exploratory measure of driving performance during the commute home, without increased sleep pressure. During the previous night shift (night shift 1), participants experienced 28 hours of extended wake by the end of the night shift to simulate the conditions commonly reported by shift workers (17). Participants completed simulated night shifts from 20:00–06:00 hours, with seven-hour daytime sleep opportunities from 10:00–17:00 hours. During night shift 2, participants in all groups underwent driving performance assessments at 20:00, 22:30, 01:30, 04:00, and 07:30 hours. During the night shift, participants also had free time to read, watch movies, play board games, and interact with other participants and laboratory staff, however no vigorous activity was allowed.

To ensure there were no differences between conditions for sleep quality and quantity, sleep was objectively recorded using polysomnography for the baseline sleep, daytime sleep after night shift 3, and the recovery sleep. Recording used Compumedics Grael EEG amplifier and acquisition software (Compumedics Ltd. Melbourne, Australia). Standard polysomnography (PSG) electrode placements were used (F3, F4, C3, C4, O1 and O2 sites), referenced to a contralateral mastoid (M1, M2). Polysomnography (PSG) data were analyzed using Rechtschaffen & Kales sleep stage scoring criteria (18). Additionally, participants wore activity monitors (Actiwatch 2, Philips Respironics Inc, Bend, OR, USA) during each sleep period.

### Eating conditions

Participants were randomized at the run-level (in groups of 3–4) to one of three eating conditions: meal at night (MN), snack at night (SN), no eating at night (NE) (table 1). Estimated energy requirement (EER) was calculated for each participant and daily 24-hour macronutrient content was consistent with the average Australian diet and standardized to approximately 40% carbohydrate, 33% fat, 17% protein, and 23g of fibre per 24 hours (Australian Bureau of Statistics 1997). For an example meal plan, see Gupta et al (16).

**Table 1:** Timing of food consumption and the percentage of estimated energy requirements (EER) for the meal, snack, and no eating at night conditions. **Bold indicates** meal or snack that was consumed during the nightshift (no food was consumed during the nightshift in the no eating at night condition).

Condition	Type of meal	Time (hours)	% EER
Meal at night	Dinner meal	19:00	40
	**Lunch-type meal**	**00:30**	**30**
	Breakfast meal	~07:00 a	30
Snack at night	Dinner meal	19:00	40
	**Snack**	**00:30**	**10**
	Breakfast meal	~07:00 a	30
	Snack	17:00	20
No eating at night	Dinner meal	19:00	40
	Breakfast meal	~07:00 a	30
	Snack	09:30	10
	Snack	17:00	20

a Breakfast time approximately 07:00 hours. The timing of the breakfast meal ranged from 06:15–07:00 hours due to metabolic testing requirements as part of the larger study. Participants had 45 minutes to consume dinner, 30 minutess to consume the lunch-type meal and snacks, and 15 minutes to consume breakfast.

During each night shift of the simulated shiftwork protocol, participants in the MN condition consumed meals at 19:00 (dinner), 00:30 (lunch-type meal) and 06:15–07:00 (breakfast) hours. These were 40%, 30% and 30% of 24-hour energy intake, respectively. The time of breakfast ranged from 06:15–07:00 hours because of metabolic testing as part of the larger study. The SN condition consumed meals and snacks at 19:00 (dinner), 00:30 (snack), 06:15–07:00 (breakfast) and 17:00 (snack) hours. These were 40%, 10%, 30% and 20% of 24-hour energy intake respectively. The NE condition consumed meals and snacks at 19:00 (dinner), 06:15–07:00 (breakfast), 09:30 (snack) and 17:00 (snack) hours. These were 40%, 30%, 10% and 20% of 24-hour energy intake respectively. Participants had 45 minutes to consume dinner, 30 minutes to consume the lunch-type meal and snacks, and 15 minutes to consume breakfast. At 00:30 hours, the NE condition were able to continue with free time activities such as watching tv, and no vigorous activity was allowed.

### Driving performance

Driving performance was assessed using a 40-minute York highway driving simulator task (York Computer Technologies, Kingston, ON, for further details please see Gupta et al (15)]. Variables used for analysis were time spent in the safe zone (SZ) (percentage of time spent within 10 km/h of the speed limit and within 0.8 m of the lane center), speed variability (SV, km/h), and lane variability (LV, m).

### Statistical analyses

Analyses were conducted with the researchers blinded to condition. Analyses were conducted using SPSS 22.00 (IBM Corp, Armonk, NY, USA). Statistical significance was defined as P<0.05. A final sample of N=39 (MN: N=12; SN: N=13; and NE: N=14) was analyzed, with five participants excluded due to a failure to comply with driving simulator instructions. Analyses of time spent outside of the SZ, SV, and LV were conducted using mixed-effects ANOVA with fixed effects of condition (MN, SN or NE), time of drive (20:00, 22:30, 01:30, 04:00, and 07:30 hours), time-on-task (40-minute drive split into eight 5-minute bins), all 2- and 3-way interaction effects involving condition, and a random effect of participant ID.

## Results

The sample had a mean age of 24.5 (SD 5.0) years, mean BMI of 23.4 (SD 2.3) kg/m^2^, and a mean 24-hour energy requirement of 9406.2 (SD 927.1) kJ. There were no significant differences between conditions for age, BMI, or average 24-hour energy intake (16). As previously published, there were no differences between groups for sleep quality or quantity (15, 16).

**Figure 2:**
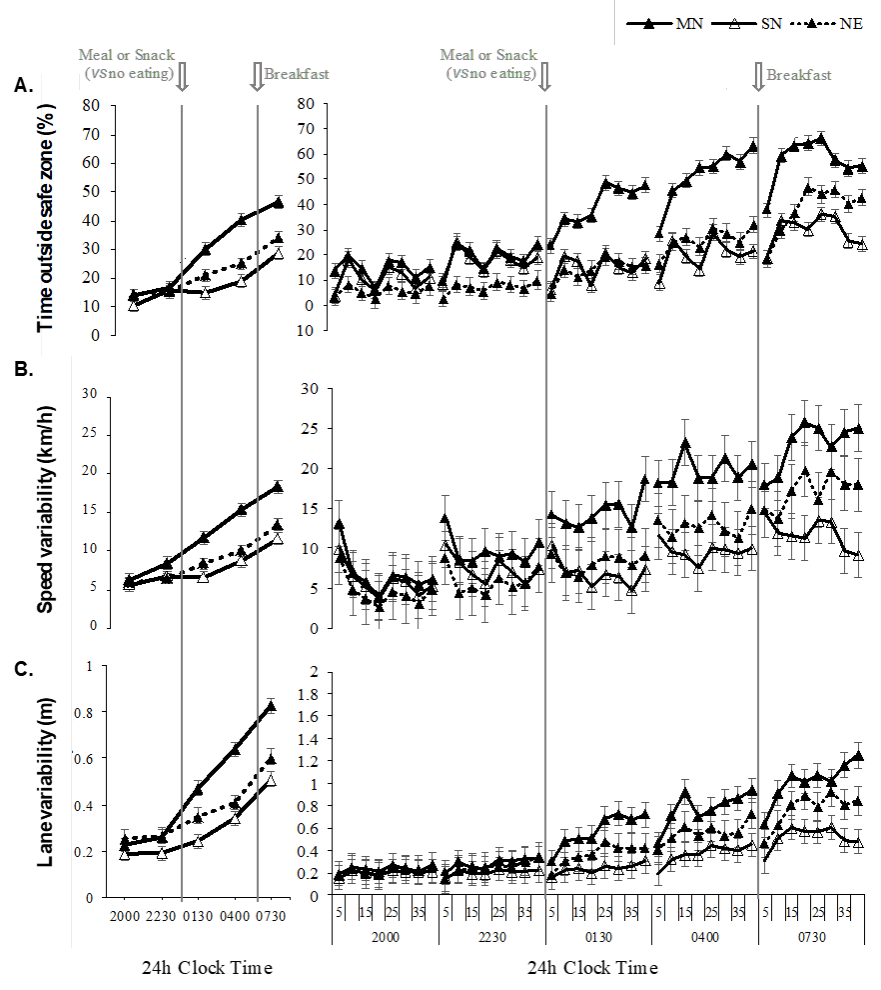
Graphs in the left column show the two-way interaction between eating condition (MN: Meal at night, SN: snack at night and NE: no eating) and performance time (20:00, 22:30, 01:30, 04:00, and 07:30 hours), and graphs in the right column show the three-way interaction between eating condition, performance time, and time-on-task (5-min bins). Panel A is time spent outside the safe zone (%), panel B is speed variability (km/h), and panel C is lane variability(m). Means presented are estimated marginal means and error bars indicate standard error from model estimates. Upwards on the y-axis indicates worse driving performance. The arrows indicate when the meal or snack was consumed (00:30 hours) and the breakfast meal was consumed by all participants (approximately 07:00 hours).

The two-way interaction between eating condition and performance time was significant for SZ [*F*(8, 934.01)=17.91, P<0.001], SF [*F*(8, 934.01)=8.72, P<0.001], and LV [*F*(8, 934.00)=13.58, P<0.001]. Post hoc analyses revealed no differences between the 20:00- and 22:30-hour drives, however driving performance worsened across the simulated night shift, with greater impairments seen at 01:30, 04:00, and 07:30 hours in the meal compared to the snack and no eating conditions (figure 2). No differences were found between the snack and the no eating condition at 07:30 hours.

The two-way interaction between condition and time-on-task was not significant for SZ [*F*(8, 934.01)=17.91, P=0.988], SV [*F*(8, 934.01)=8.72, P=<0.001], or LV [*F*(8, 934.00)=13.58, P<0.001]. The three-way interaction between condition, performance time and time-on-task was not significant for SZ [*F*(56, 934.00)=0.45, P=1.00], SV [*F*(56, 934.00)=0.38, P=1.00], or LV [*F*(56, 934.00)=0.35, P=1.00].

The main effect of drive was significant for SZ [*F*(4, 934.01)=151.43, P<0.001], SV [*F*(4, 934.01)=95.51, P<0.001], and LV [*F*(4, 934.00)=177.80, P<0.001], and the main effect of time-on-task was significant for SZ [*F*(7, 934.00)=13.45, P<0.001], SV [*F*(7, 934.00)=2.55, P=0.01], and LV [*F*(7, 934.01)=12.03, P<0.001]. The main effect of condition was significant for SZ [*F*(2, 24.00)=3.62, P=0.04], but not significant for SV [*F*(2, 24.00)=3.11, P=0.06] or LV [*F*(2, 24.00)=3.26, P=0.06].

## Discussion

This study investigated the post-prandial impact on driving performance at the end of a simulated night shift, following consumption of a meal. Driving performance followed the expected pattern of impairment, worsening across the night from 01:30–07:30 hours and with increasing time-on-task, regardless of eating condition (1, 19). This was exacerbated for those who consumed the meal compared to the snack or ate no food during the night. Those who consumed the meal were still significantly more impaired during the commute home than those who consumed a snack and those who did not eat during the night. This provides preliminary evidence that reducing the amount of food consumed during the night shift may be a modifiable factor to improve safety on the consume home. Additionally, we have previously found that while those who consumed a snack during the night shift did not report feeling full across the night shift, they did not report a greater desire to eat than those who consumed the meal (16). This suggests that the recommendation to consume a snack during the night shift may be a feasible option as workers may be satisfied with this amount of food.

The difference in driving performance between conditions may be influenced by subjective sleepiness. Previously published findings from this study have shown increased subjective sleepiness across the night shift, with greater sleepiness at 04:00 compared to 20:00 hours (15). Further, the greatest increase in subjective sleepiness was reported by those who had consumed the meal during the night shift compared to the snack (15). While subjective sleepiness was not recorded immediately prior to the commute home, a reason for the driving impairment during the commute among those who consumed the meal may be greater subjective sleepiness as a result of the meal at 00:30 hours. In addition to increased sleepiness, there are several suggested mechanisms for the post-prandial impairment found after eating a large meal during the biological night. Reduced glucose tolerance (20) and reduced rates of gastric emptying (21) at night may cause the meal to be a greater challenge to the digestive system compared to snacking or not eating. Further, performing cognitive tasks at night requires a redistribution of blood flow (22), and, given that brain resources are also required to digest the meal, these competing demands may lead to cognitive impairment.

In contrast to eating during the night, the breakfast meal did not further impact performance beyond the increasing pattern of impairment in which the data were already trending. This study is limited in that we do not have a ‘no breakfast’ comparison in order to specifically quantify the impact of the breakfast, and that the between-subjects nature of the experimental groups (despite random assignment) leaves an open question as to the potential contribution of individual differences to the observed patterns. Given that the breakfast was the same size as the meal at night, the minimal impact of the breakfast on driving performance may further reinforce the importance of considering the timing of meals. That is, it could be (very tentatively) argued from these data that eating a meal of the same size during the biological night (at 00:30 hour) may have a larger impact on the post-shift commute than eating in the morning before the drive (06:15–07:00 hours).

Overall, results support the value of researching the possibility of changing meal size and timing as a countermeasure for night shift workers, however there are limitations to these results that must be considered. While the controlled laboratory environment was a necessary first step to investigate post-prandial effects on performance, the controlled lighting, temperature, time cues, and sound may have increased sleep quality and quantity in comparison to real-world shift workers who must contend with external factors such as environmental noise and outside light (17). This reduced sleep may worsen cognitive performance during the commute home, due to increasing sleep pressure (17) and may influence the post-prandial response (23). A further limitation to consider is that the participants in the study were young, healthy, and had no shift working experience. This does limit generalizability to shiftwork populations, which may include those with a higher risk of conditions that may worsen the post-prandial response, such as health issues such as obesity and metabolic disorders (23, 24). Further, this exploratory analysis used a low fidelity driving simulator, with minimal traffic and a speed limit of 100 km/hour. While this was chosen as a measure of sustained attention in monotonous conditions, shift workers in the real-world may be faced with factors such as reduced visibility and increased traffic during the commute home (25). Additionally, potential between-group differences in uncontrolled variables could potentially help to explain some of the findings. However, given randomized group allocation and no evidence of any relevant differences between groups (15), this appears unlikely.

The next steps include systematic investigation of different eating patterns to quantify the potential impacts of all pre-commute eating occasions (especially breakfast), varying macronutrient content, combinations of other countermeasures (eg, snack plus caffeine), and objective measures of alertness and sleepiness before and during the drive, as well as moving to field trials in shift working populations with different health profiles to our laboratory participants (24). A further suggestion for future investigation is the cumulative impact of food intake on post-prandial performance during the commute home across multiple night shifts. Although we did not find an impact on driving performance during the night shift after four consecutive night shifts (15), perhaps after an increased number of consecutive shifts with reduced sleep intake between shifts commonly reported for shift workers (17), we would see a cumulative effect.

The present study showed that under simulated conditions, a snack during the night shift is a better option for post-shift driving performance compared to larger amounts of food. Future research should continue to explore food intake during shift work as a potential fatigue countermeasure in real-life settings among shift working populations.
